# Human T-cell lymphotropic virus type 1 (HTLV-1) infection among couples of a cohort followed up in São Paulo, Brazil

**DOI:** 10.1186/1742-4690-12-S1-P34

**Published:** 2015-08-28

**Authors:** Arthur Paiva, Tatiane Assone, Samara SP Gomes, Jerusa Smid, Augusto Penalva de Oliveira, Jorge Casseb

**Affiliations:** 1 Instituto de Medicina Tropical de São Paulo, Universidade de São Paulo, São Paulo, Brazil; 2 Instituto de Infectologia “Emílio Ribas”, São Paulo, Brazil

## 

This study proposes to investigate the vertical and sexual HTLV-1 transmission rate in a coorte followed up in São Paulo, Brazil. The latter 173 HTLV-1-infected patients were selected until july 2014 (27.2% of 636 HTLV-1-infected individuals). Data were recorded using RedCap (Research Electronic Data Capture), transported to SPSS – Statistical Package for Social Sciences (v 20) and subjected to statistical analysis. To compare results between groups, one-way analysis of variance (one-way ANOVA test) was used. The vertical transmission rate found among 73 individuals born to HTLV-1-positive mothers was 6.8%. HTLV-1 concordant couples were compared to HTLV-1 discordant couples. Those coinfected with HIV were excluded from the sample. Among 91 individuals with stable partnership and without HIV, 49% did not know about the current partner serology: 39% of wives and 54.5% of husbands have not been tested. The horizontal transmission rate of HTLV-1 among those tested couples was 55.3%. Among men 68.2% of its wives were seropositive for HTLV-1, while among women 44.0% of its husbands were seropositive. HTLV-1 concordant couples (cases, n=6) were also compared to HTLV-1 discordant couples (controls, n=14), with respect to HTLV-1 proviral load. Those without HTLV-1 proviral load were excluded. In turn, the control group was subdivided into two subgroups: discordant couples with male index partner (n=5) and discordant couples with female index partner (n=9). Because of the impossibility of defining whether sexual transmission had occurred from man to woman or woman to man, for group 1 (concordant couples) we chose to use the average of the proviral loads of both partners in this group. Serodiscordant couples showed higher mean proviral loads (670 ±515 cópias/104 peripheral blood mononuclear cell (PBMC)) compared with serodiscordant couples (282 ± 293 cópias/104 PBMC) (p=0.045), probably associated with increased genital shedding of this virus and resulting increased risk of sexual transmission.

